# PopGLen—a Snakemake pipeline for performing population genomic analyses using genotype likelihood-based methods

**DOI:** 10.1093/bioinformatics/btaf105

**Published:** 2025-03-11

**Authors:** Zachary J Nolen

**Affiliations:** Department of Biology, Lund University, Lund 22 362, Sweden

## Abstract

**Summary:**

PopGLen is a Snakemake workflow for performing population genomic analyses within a genotype-likelihood framework, integrating steps for raw sequence processing of both historical and modern DNA, quality control, multiple filtering schemes, and population genomic analysis. Currently, the population genomic analyses included allow for estimating linkage disequilibrium, kinship, genetic diversity, genetic differentiation, population structure, inbreeding, and allele frequencies. Through Snakemake, it is highly scalable, and all steps of the workflow are automated, with results compiled into an HTML report. PopGLen provides an efficient, customizable, and reproducible option for analyzing population genomic datasets across a wide variety of organisms.

**Availability and implementation:**

PopGLen is available under GPLv3 with code, documentation, and a tutorial at https://github.com/zjnolen/PopGLen. An example HTML report using the tutorial dataset is included in the Supplementary Material.

## 1 Introduction

Genomic resources are rapidly becoming available for non-model organisms due to efforts to generate reference genomes for the majority of the planet’s species. These genomes make population genomic analyses possible for many wild species for the first time, addressing an increasing need for genetic estimates to address research questions and inform conservation action. However, the data types that go into these studies are not always suited for traditional genotype call based population genomics, in particular for studies with low sequencing depth (Lou *et al.* 2021), such as those utilizing degraded DNA from historical samples (Díez-del-Molino *et al.* 2018). Genotype likelihood-based methods are especially suited for these data types, utilizing a probabilistic framework that incorporates the uncertainty in base calling (Nielsen *et al.* 2011).

**Figure 1.**
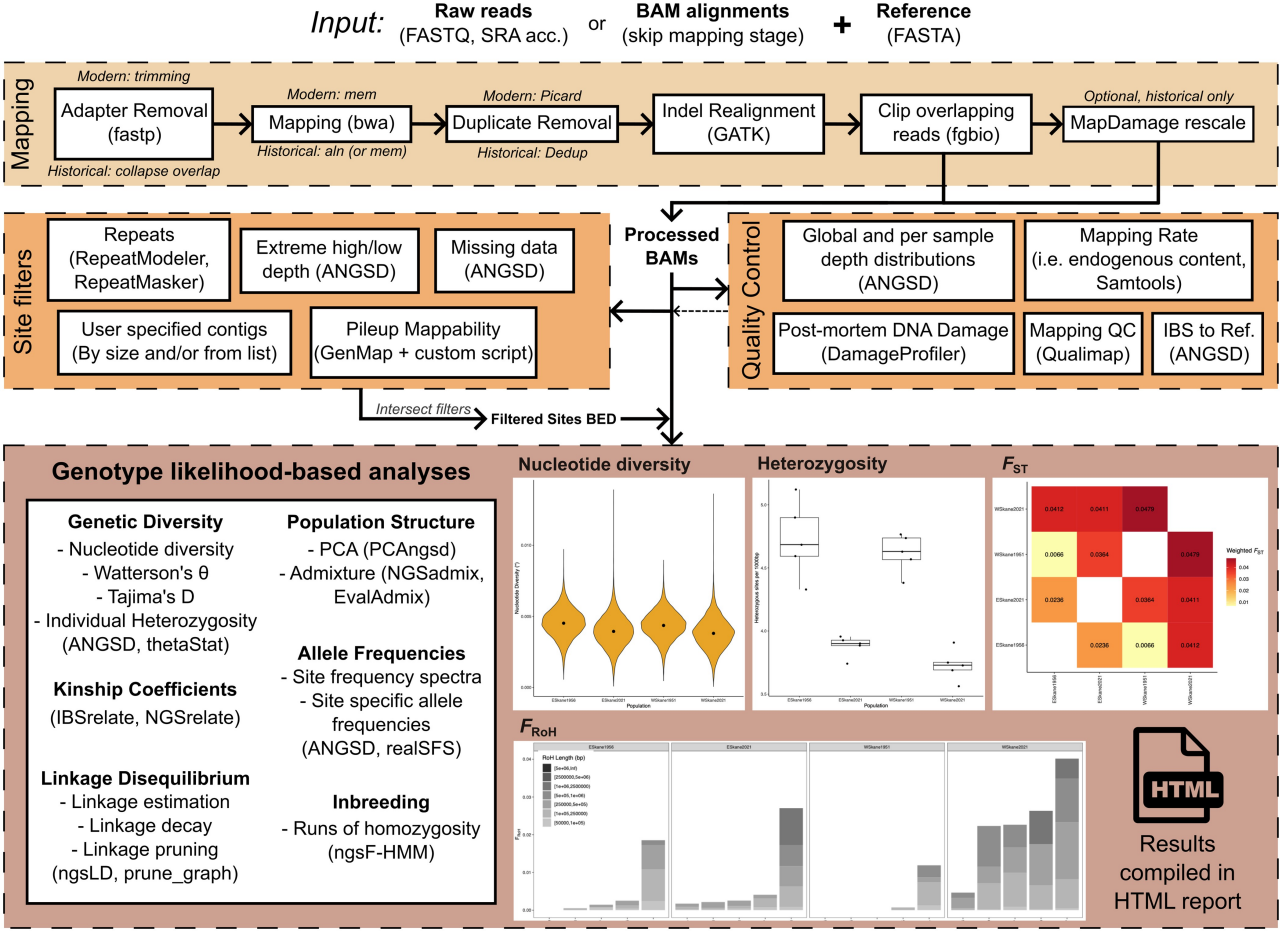
A broad overview of the workflow, analyses, and reporting available with PopGLen. Input requires a reference genome and sequencing reads provided as raw reads (FASTQ) or alignments (BAM). The workflow is divided into four modular components (dashed boxes): (i) mapping of sequence data to the reference genome (omitted for BAM input) with separate pathways for historical and modern samples, (ii) generation of a filtered sites file to limit analyses to, (iii) quality control to assess data quality and inform configuration, and (iv) population genomic analyses using genotype likelihood-based tools. Within each component, analyses are individually enabled and configured, allowing for high customizability. Once configured, all steps in the pipeline are automated using Snakemake and can be run using a single command. Figures and tables are prepared for most analyses and included in an HTML report. Four example plots are included here, based on data in the tutorial dataset, which examines declines in genetic diversity and increases in genetic differentiation and inbreeding in a pair of modern butterfly populations compared to their historical counterparts. The example plots have been resized but remain otherwise unmodified from the pipeline output. Higher resolution versions and other example outputs can be viewed in the example report included in the Supplementary Material.

Here, I describe PopGLen, a pipeline I have developed to be an all-inclusive workflow that includes raw data processing and mapping, quality control, multiple filtering schemes, and a wide breadth of genotype likelihood-based analyses (Fig. 1). It is developed using the Snakemake workflow manager (Mölder *et al.* 2021), enabling it to be easily deployable, scalable, modular, and reproducible. Other workflows partially overlap these aims, such as processing historical DNA sequencing data (PALEOMIX—Schubert *et al.* 2014, nf-core/eager—Peltzer *et al.* 2016, Mapache—Neuenschwander *et al.* 2023), analysis of combined historical and modern DNA datasets (GenErode—Kutschera *et al.* 2022), and running genotype likelihood analyses from BAMs (loco-pipe—Zhou *et al.* 2024, see Supplementary Tables S1 and S2 for a comparison of PopGLen’s features with these pipelines). PopGLen aims to incorporate all necessary steps to process raw sequencing data into population genomic results in a way that is flexible to datasets with both modern and historical DNA by performing alternate processing and filtering when required. To enable freely combining PopGLen with related workflows, including those cited above, the pipeline contains several modular components (Fig. 1), allowing users to substitute and extend portions of the workflow to their liking.

## 2 Approach and features

### 2.1 Preparation and input data

Sample data is required as either raw FASTQ files or aligned BAM files, and a local copy of the reference genome must be provided. Configuration is handled with (i) a YAML file where analyses are enabled and software configurations are set, (ii) a sample metadata list, assigning samples to populations, and (iii) a sequencing unit list, which links sample IDs to their input files. In the config file a name is given to the dataset, and all results will be placed in a folder under that name, allowing for multiple datasets to be managed within one working directory where they share relevant intermediate files (e.g. BAMs, repeat libraries).

### 2.2 Data processing

#### 2.2.1 Reference genome preparation

To enable parallel processing, the pipeline groups contigs into similar sized “chunks” of a user-defined size, scalable to many thousands of contigs. Mitochondrial, sex-linked, and other specified contigs can be excluded from these chunks.

#### 2.2.2 Raw sequence data processing

Raw sequence data input in FASTQ format can be provided either as local paths or NCBI SRA accessions, the latter being downloaded automatically. Raw reads are processed and aligned to the reference genome for samples provided as raw reads rather than aligned BAMs.

Modern samples have adapters trimmed with fastp (Chen *et al.* 2018) and trimmed paired-end reads are mapped to the reference using BWA-MEM (Li 2013). If multiple libraries are provided for a sample, the subsequent BAM files are merged with Samtools (Danecek *et al.* 2021). Afterwards, duplicates are removed with Picard MarkDuplicates, reads are realigned around indels with GATK IndelRealigner (Van der Auwera and O’Connor 2020), and overlapping reads are trimmed by BamUtil (Jun *et al.* 2015) to avoid double counting of overlapping reads by ANGSD (Lou *et al.* 2021).

Historical samples will have pairs of overlapping reads collapsed by fastp, generating single reads. By default, collapsed reads are mapped using BWA-ALN using settings optimized for historical samples (Palkopoulou *et al.* 2015). Duplicates are removed with DeDup (Peltzer *et al.* 2016), which considers both read ends of the read. Reads are realigned around indels with GATK IndelRealigner and, optionally, base quality scores can be recalibrated using MapDamage 2 (Jónsson *et al.* 2013) to account for post-mortem DNA damage.

#### 2.2.3 Data filtering

A sites-based filtering scheme is used to limit analyses to suitable sites (adapted from e.g. Pečnerová *et al.* 2021, Quinn *et al.* 2023). Each site filter is optional and generated independently. All filters are intersected using BEDTools (Quinlan and Hall 2010) to create a final usable sites list. Filters for mappability, repetitive content, extreme high or low global sequencing depth and missing data are included. Users can provide additional filters as BED files e.g. to restrict analyses to neutral sites or genic regions. The proportion of the genome passing each filter, and the combined filter set, is described in a summary table.

Low mappability regions are identified using mappability scores from GenMap (Pockrandt *et al.* 2020). These scores are converted to pileup mappability (Derrien *et al.* 2012), by averaging the mappability score of all fragments overlapping a position using BedOps (Neph *et al.* 2012). Both the fragment size (K) and allowed mismatches (E) used to estimate scores in GenMap, and the pileup mappability threshold, are set in the config file.

Repeat content identification and filtering is implemented through (i) building of a de-novo repeat library with RepeatModeler (Flynn *et al.* 2020), (ii) using a provided repeat library informing repetitive region identification with RepeatMasker (Smit *et al.* 2013), or (iii) removing repetitive regions provided in a BED/GFF file.

Upper and lower global depth filters are calculated across all samples, as well as for user-defined subsets of samples if configured. A filtering threshold can either be defined using upper/lower percentiles or multipliers to the median global sequencing depth.

#### 2.2.4 Quality control

Several metrics to assess quality are compiled into the pipeline report. Adapter trimming reports are directly provided by fastp. Mapping rates are provided using Samtools. ANGSD is used to calculate mean and standard deviation sequencing depth per sample for (i) all positions without read filtering, (ii) all positions passing mapping and base quality thresholds, (iii) positions passing the main filter set, and (iv) positions passing any user-provided filter sets. Post-mortem DNA damage reports are provided by MapDamage2 and/or DamageProfiler (Neukamm *et al.* 2021). Reports containing general mapping statistics such as average mapping quality and GC content are provided by Qualimap (García-Alcalde *et al.* 2012). Identity by state similarity to the reference is estimated using ANGSD.

### 2.3 Population genomic analyses

Each of the analyses described below can be individually enabled in the config file. Analyses utilize genotype likelihoods for SNPs in Beagle format or site allele frequencies in SAF format, which are generated as needed and shared between analyses where appropriate. Transition removal in ANGSD to account for post-mortem DNA damage can be enabled across all analyses in the configuration file.

Estimation of linkage disequilibrium (LD) and LD decay are available using ngsLD (Fox *et al.* 2019), both across the whole dataset and for each population separately. Before running analyses that assume independence of positions, LD estimates are used to prune SNPs using prune_graph (https://github.com/fgvieira/prune_graph).

Two approaches for illustrating and assessing population structure are implemented—principal component analysis (PCA) using PCAngsd (Meisner and Albrechtsen 2018) and admixture analysis using NGSadmix (Skotte *et al.* 2013), both based on pruned SNPs. For each value of K, multiple independent NGSadmix replicates are performed to assess convergence, following customizable criteria described by Pečnerová *et al.* (2021). EvalAdmix (Garcia-Erill and Albrechtsen 2020) is run to provide an additional assessment of model fit. A list of individuals to exclude from these two analyses can be provided in the config to enable removing close relatives.

Kinship is inferred using IBSrelate (Waples *et al.* 2019) and/or ngsRelateV2 (Hanghøj *et al.* 2019). For IBSrelate, population allele frequencies are not required and both the IBS-based method implemented in ANGSD and the SFS-based method implemented in both ANGSD and ngsRelateV2 can be performed for all pairs of samples, with results provided in a table of R0, R1, and KING-robust kinship coefficients. The allele frequency-based methods in ngsRelateV2 can additionally be performed on each population, with allele frequencies estimated automatically by ANGSD and used by ngsRelateV2.

As a basis for population genomic estimates, folded and unfolded site frequency spectra (SFS) for single populations and population pairs are produced by ANGSD as needed for downstream analyses or as a requested output. Bootstrapped SFS can be additionally estimated, with the bootstrap count defined in the config file. Site frequency spectra are polarized to the reference allele, unless ancestral states are provided.

The pipeline has various options for estimating genetic diversity and neutrality statistics using ANGSD. Per-population estimates are performed in sliding windows, using user-defined size and step. Pairwise nucleotide diversity (π), Watterson’s estimator ($\theta_{W}$) and Tajima’s D are also summarized into genome-wide means and confidence intervals per population. Individual heterozygosity is estimated from a single sample SFS by dividing the number of heterozygous positions by the total number of sites using ANGSD. Confidence intervals are inferred using the bootstraps of the single sample SFS.

Genetic differentiation, pairwise FST, is estimated both globally and in windows using ANGSD for all population pairs. The estimator used can be set in the config file, with the default set to the Hudson-Bhatia estimator (Bhatia *et al.* 2013), which is suited for small sample sizes.

Inbreeding coefficients are estimated from identical by descent (IBD) tracts using ngsF-HMM (Vieira *et al.* 2016). IBD tracts are estimated for pruned SNPs called within each population. Inbreeding coefficients are estimated both by ngsF-HMM and using the inferred IBD tracts to estimate $F_{\mathrm{RoH}}$, the proportion of the autosomal genome covered by runs of homozygosity greater than a user-defined length (McQuillan *et al.* 2008).

An identity by state (IBS) matrix between all samples is estimated using SNPs called across the dataset in ANGSD.

Population allele frequencies are estimated using ANGSD. Each population has allele frequencies estimated for (i) variable sites within the population, assigning the minor allele to the population specific minor and (ii) variable sites within the dataset, including invariable sites within the population, assigning the minor allele to the minor of the entire dataset. Alternatively, the “major” allele can be fixed to the reference or provided ancestral allele.

Variation in sequencing depth can influence the outputs of many analyses. Users can define one or more target subsampled sequencing depths to subsample all samples to using Samtools. Each analysis can be separately enabled to run on subsampled data, allowing for comparisons between full and subsampled depth outputs to assess the influence of sequencing depth variation.

### 2.4 Reporting

This pipeline uses Snakemake’s native report features to compile the results of the pipeline into a single HTML report. Quality control statistics summarized with MultiQC (Ewels *et al.* 2016) and figures are generated for several analyses (Fig. 1) in R (R Core Team 2017). The report can be compiled for any successful run of the pipeline, allowing for easy assessment of partial runs to inform later analyses. An example report from the tutorial dataset, with all analyses enabled, is included in the Supplementary Material.

## 3 Availability, execution, and use cases

PopGLen is available on GitHub with documentation and a tutorial dataset (https://zjnolen.github.io/PopGLen) and can be deployed as a module in a single Snakefile using Snakedeploy. In this modular format, users can customize the workflow by adding additional rules that use PopGLen outputs as input, allowing extension or replacement of specific analyses. Required software are defined as Conda environments (Grüning *et al.* 2018) or Singularity containers (da Veiga Leprevost *et al.* 2017), requiring no pre-installed software aside from Conda and Singularity. After configuration, the pipeline can be run with a single Snakemake command, which will infer the required steps to generate the requested output, prepare the required software, and execute the workflow. Using Snakemake’s executor plugins, it is compatible with a variety of high-performance computing job queue systems, enabling high levels of parallelization as jobs are submitted and monitored automatically to multiple nodes. I have ensured compatibility with Snakemake’s code and parameter monitoring features, meaning changes to settings in the config or addition/removal of samples will trigger re-runs of relevant steps.

PopGLen is an efficient, flexible, and reproducible way for population genomic projects to go from raw data to several common population genomic statistics. It is particularly well suited for historical or low sequencing depth samples and comparisons of such samples to contemporary ones with higher coverage. It has applicability in a variety of research fields utilizing the implemented methods, including conservation, speciation, and evolutionary ecology.

## Supplementary Material

btaf105_Supplementary_Data

## Data Availability

PopGLen and its documentation is available at https://github.com/zjnolen/PopGLen, where questions, bug reports, and feature requests can be submitted. Releases are archived at https://doi.org/10.5281/zenodo.13384125. The data required to follow the tutorial is available at https://doi.org/10.6084/m9.figshare.27453978.v1.

## References

[btaf105-B2] Bhatia G , Patterson N, Sankararaman S et al Estimating and interpreting FST: the impact of rare variants. Genome Res 2013;23:1514–21. 23861382 10.1101/gr.154831.113 PMC3759727

[btaf105-B3] Chen S , Zhou Y, Chen Y et al Fastp: an ultra-fast all-in-one FASTQ preprocessor. Bioinformatics 2018;34:i884–90. 30423086 10.1093/bioinformatics/bty560 PMC6129281

[btaf105-B4] da Veiga Leprevost F , Grüning BA, Alves Aflitos S et al BioContainers: an open-source and community-driven framework for software standardization. Bioinformatics 2017;33:2580–2. 28379341 10.1093/bioinformatics/btx192 PMC5870671

[btaf105-B5] Danecek P , Bonfield JK, Liddle J et al Twelve years of SAMtools and BCFtools. Gigascience 2021;10:1–4. 10.1093/gigascience/giab008 PMC7931819 33590861

[btaf105-B6] Díez-Del-Molino D , Sánchez-Barreiro F, Barnes I et al Quantifying temporal genomic erosion in endangered species. Trends Ecol Evol 2018;33:176–85. 29289355 10.1016/j.tree.2017.12.002

[btaf105-B7] Derrien T , Estellé J, Marco Sola S et al Fast computation and applications of genome mappability. PLoS One 2012;7:e30377. 22276185 10.1371/journal.pone.0030377 PMC3261895

[btaf105-B8] Ewels P , Magnusson M, Lundin S et al MultiQC: summarize analysis results for multiple tools and samples in a single report. Bioinformatics 2016;32:3047–8. 27312411 10.1093/bioinformatics/btw354 PMC5039924

[btaf105-B9] Flynn JM , Hubley R, Goubert C et al RepeatModeler2 for automated genomic discovery of transposable element families. Proc Natl Acad Sci USA 2020;117:9451–7. 32300014 10.1073/pnas.1921046117 PMC7196820

[btaf105-B10] Fox EA , Wright AE, Fumagalli M et al ngsLD: evaluating linkage disequilibrium using genotype likelihoods. Bioinformatics 2019;35:3855–6. 30903149 10.1093/bioinformatics/btz200

[btaf105-B11] García-Alcalde F , Okonechnikov K, Carbonell J et al Qualimap: evaluating next-generation sequencing alignment data. Bioinformatics 2012;28:2678–9. 22914218 10.1093/bioinformatics/bts503

[btaf105-B12] Garcia-Erill G , Albrechtsen A. Evaluation of model fit of inferred admixture proportions. Mol Ecol Resources 2020;20:936–49. 10.1111/1755-0998.13171 32323416

[btaf105-B13] Grüning B , Dale R, Sjödin A et al; Bioconda Team. Bioconda: sustainable and comprehensive software distribution for the life sciences. Nat Methods 2018;15:475–6. 29967506 10.1038/s41592-018-0046-7 PMC11070151

[btaf105-B14] Hanghøj K , Moltke I, Andersen PA et al Fast and accurate relatedness estimation from high-throughput sequencing data in the presence of inbreeding. Gigascience 2019;8:giz034. 31042285 10.1093/gigascience/giz034 PMC6488770

[btaf105-B15] Jónsson H , Ginolhac A, Schubert M et al mapDamage2.0: fast approximate Bayesian estimates of ancient DNA damage parameters. Bioinformatics 2013;29:1682–4. 23613487 10.1093/bioinformatics/btt193 PMC3694634

[btaf105-B16] Jun G , Wing MK, Abecasis GR et al An efficient and scalable analysis framework for variant extraction and refinement from population-scale DNA sequence data. Genome Res 2015;25:918–25. 25883319 10.1101/gr.176552.114 PMC4448687

[btaf105-B17] Kutschera VE , Kierczak M, van der Valk T et al GenErode: a bioinformatics pipeline to investigate genome erosion in endangered and extinct species. BMC Bioinformatics 2022;23:228–17. 35698034 10.1186/s12859-022-04757-0 PMC9195343

[btaf105-B18] Li H. Aligning sequence reads, clone sequences and assembly contigs with BWA-MEM. arXiv 2013; 1303.3997.

[btaf105-B19] Lou RN , Jacobs A, Wilder AP et al A beginner’s guide to low-coverage whole genome sequencing for population genomics. Mol Ecol 2021;30:5966–93. 34250668 10.1111/mec.16077

[btaf105-B20] McQuillan R , Leutenegger A-L, Abdel-Rahman R et al Runs of homozygosity in European populations. Am J Hum Genet 2008;83:359–72. 18760389 10.1016/j.ajhg.2008.08.007 PMC2556426

[btaf105-B21] Meisner J , Albrechtsen A. Inferring population structure and admixture proportions in low-depth NGS data. Genetics 2018;210:719–31. 30131346 10.1534/genetics.118.301336 PMC6216594

[btaf105-B22] Mölder F , Jablonski KP, Letcher B et al Sustainable data analysis with snakemake [version 2; peer review: 2 approved]. F1000Res 2021;10:33. 34035898 10.12688/f1000research.29032.1 PMC8114187

[btaf105-B23] Neph S , Kuehn MS, Reynolds AP et al BEDOPS: high-performance genomic feature operations. Bioinformatics 2012;28:1919–20. 22576172 10.1093/bioinformatics/bts277 PMC3389768

[btaf105-B24] Neuenschwander S , Cruz Dávalos DI, Anchieri L et al Mapache: a flexible pipeline to map ancient DNA. Bioinformatics 2023;39:btad028. 36637197 10.1093/bioinformatics/btad028 PMC9901408

[btaf105-B25] Neukamm J , Peltzer A, Nieselt K et al DamageProfiler: fast damage pattern calculation for ancient DNA. Bioinformatics 2021;37:3652–3. 33890614 10.1093/bioinformatics/btab190

[btaf105-B26] Nielsen R , Paul JS, Albrechtsen A et al Genotype and SNP calling from next-generation sequencing data. Nat Rev Genet 2011;12:443–51. 21587300 10.1038/nrg2986 PMC3593722

[btaf105-B27] Palkopoulou E , Mallick S, Skoglund P et al Complete genomes reveal signatures of demographic and genetic declines in the woolly mammoth. Curr Biol 2015;25:1395–400. 25913407 10.1016/j.cub.2015.04.007 PMC4439331

[btaf105-B28] Pečnerová P , Garcia-Erill G, Liu X et al High genetic diversity and low differentiation reflect the ecological versatility of the African leopard. Curr Biol 2021;31:1862–71.e5. 33636121 10.1016/j.cub.2021.01.064

[btaf105-B29] Peltzer A , Jäger G, Herbig A et al EAGER: efficient ancient genome reconstruction. Genome Biol 2016;17:60–14. 27036623 10.1186/s13059-016-0918-z PMC4815194

[btaf105-B30] Pockrandt C , Alzamel M, Iliopoulos CS et al GenMap: ultra-fast computation of genome mappability. Bioinformatics 2020;36:3687–92. 32246826 10.1093/bioinformatics/btaa222 PMC7320602

[btaf105-B31] Quinlan AR , Hall IM. BEDTools: a flexible suite of utilities for comparing genomic features. Bioinformatics 2010;26:841–2. 20110278 10.1093/bioinformatics/btq033 PMC2832824

[btaf105-B32] Quinn L , Garcia-Erill G, Santander C et al Colonialism in South Africa leaves a lasting legacy of reduced genetic diversity in Cape buffalo. Mol Ecol 2023;32:1860–74. 36651275 10.1111/mec.16851

[btaf105-B33] R Core Team. R: A Language and Environment for Statistical Computing. Vienna, Austria: R Foundation for Statistical Computing, 2017.

[btaf105-B34] Schubert M , Ermini L, Der Sarkissian C et al Characterization of ancient and modern genomes by SNP detection and phylogenomic and metagenomic analysis using PALEOMIX. Nat Protoc 2014;9:1056–82. 24722405 10.1038/nprot.2014.063

[btaf105-B35] Skotte L , Korneliussen TS, Albrechtsen A et al Estimating individual admixture proportions from next generation sequencing data. Genetics 2013;195:693–702. 24026093 10.1534/genetics.113.154138 PMC3813857

[btaf105-B36] Smit A FA, Hubley R, Green P. *RepeatMasker Open-4.0*. 2013–2015. http://www.repeatmasker.org.

[btaf105-B1] Van der Auwera GA , O’Connor BD. Genomics in the Cloud: Using Docker, GATK, and WDL in Terra. Sebastopol, CA, USA: O’Reilly Media, Inc. 2020.

[btaf105-B37] Vieira FG , Albrechtsen A, Nielsen R et al Estimating IBD tracts from low coverage NGS data. Bioinformatics 2016;32:2096–102. 27153648 10.1093/bioinformatics/btw212

[btaf105-B38] Waples RK , Albrechtsen A, Moltke I et al Allele frequency-free inference of close familial relationships from genotypes or low-depth sequencing data. Mol Ecol 2019;28:35–48. 30462358 10.1111/mec.14954 PMC6850436

[btaf105-B39] Zhou ZT , Owens GL, Larson WA et al loco-pipe: an automated pipeline for population genomics with low-coverage whole-genome sequencing. Bioinform Adv 2024;4:vbae098. 39006965 10.1093/bioadv/vbae098 PMC11246161

