# Supplementary material for: PopGLen—a Snakemake pipeline for performing population genomic analyses using genotype likelihood-based methods
Source: Bioinformatics. 2025 Mar 11;41(3):btaf105. doi: 10.1093/bioinformatics/btaf105 (PMC11932725; doi:10.1093/bioinformatics/btaf105)
Supplement: btaf105_Supplementary_Data [file btaf105_supplementary_data.zip › popglen-supplementary-example-report.html]

Snakemake Report


Loading Snakemake Report...

Please enable Javascript in your browser to see this report.

# SnakemakeReport

- General
- Workflow
- Statistics
- About
- Result
- 00 Quality Control
- 01 Linkage Disequilibrium Decay
- 02 Relatedness
- 03.1 PCA
- 03.2 Admixture
- 04.1 Watterson's Theta
- 04.2 Nucleotide Diversity (Pi)
- 04.3 Tajima's D
- 04.4 Heterozygosity
- 05 Fst
- 06 Inbreeding

Save as SVGSave as PNGView SourceOpen in Vega Editor
