# Supplementary material for: PopGLen—a Snakemake pipeline for performing population genomic analyses using genotype likelihood-based methods
Source: Bioinformatics. 2025 Mar 11;41(3):btaf105. doi: 10.1093/bioinformatics/btaf105 (PMC11932725; doi:10.1093/bioinformatics/btaf105)
Supplement: btaf105_Supplementary_Data [file btaf105_supplementary_data.zip › supplementary-tables-popglen.pdf]

Supplementary Tables

PopGLen - A Snakemake pipeline for performing population genomic analyses using genotype likelihood-based methods

Zachary J. Nolen[1]

[1.] Department of Biology, Lund University, Kontaktvägen 10, 22 362 Lund, Sweden

E-mail: zachary.nolen@biol.lu.se

Abstract

Summary: PopGLen is a Snakemake workflow for performing population genomic analyses within a genotype-likelihood framework, integrating steps for raw sequence processing of both historical and modern DNA, quality control, multiple filtering schemes, and population genomic analysis. Currently, the population genomic analyses included allow for estimating linkage disequilibrium, kinship, genetic diversity, genetic differentiation, population structure, inbreeding, and allele frequencies. Through Snakemake, it is highly scalable, and all steps of the workflow are automated, with results compiled into an HTML report. PopGLen provides an efficient, customizable, and reproducible option for analyzing population genomic datasets across a wide variety of organisms.

Availability and implementation: PopGLen is available under GPLv3 with code, documentation, and a tutorial at https://github.com/zjnolen/PopGLen. An example HTML report using the tutorial dataset is included in the supplementary material.

Table S1. Comparison of PopGLen's implementation to similar pipelines. PopGLen is aimed at users seeking an automated workflow for both raw sequence processing and genotype likelihood based population genomic analyses. Several automated workflows have been released with partial overlap, with recent pipelines, including PopGLen, using Snakemake (Mölder *et al.*, 2021) or Nextflow (Di Tommaso *et al.*, 2017) as their workflow managers. This enables them to be automated, highly scalable, compatible with high performance computing job queues, and offer software installation of required tools. Mapache (Neuenschwander *et al.*, 2023) and nf-core/EAGER (Peltzer *et al.*, 2016) both aim at processing raw historical sample sequences into alignments, as does both PALEOMIX (Schubert *et al.*, 2014) and GenErode (Kutschera *et al.*, 2022), which are also flexible to processing modern samples. Of these four, only GenErode additionally performs population genomic analyses as PopGLen does. PopGLen differs from GenErode in its focus on genotype likelihood based analyses, which are suited for low coverage data, whereas GenErode uses genotype call based analyses where a minimum average of 6X coverage is recommended. loco-pipe (Zhou *et al.*, 2024) is a recent pipeline that focuses on many of the same genotype likelihood based analyses as PopGLen, providing the most similar functionality, though it differs in that it requires separate preprocessing of the raw sequence data into alignments, which PopGLen includes. See Table S2 for a comparison of population genomic analyses available in PopGLen, loco-pipe, and GenErode.

	PopGLen	***loco-pipe***	***GenErode***	***mapache***	***nf-core/ EAGER***	***PALEOMIX***
Workflow manager	Snakemake	Snakemake	Snakemake	Snakemake	Nextflow	Built-in
Installs software	All	Requires user install of some tools	All	All	All	No
Job queue integration	Yes	Yes	Yes	Yes	Yes	No
Processes raw sequences	Yes (historical and/or modern)	No	Yes (historical and/or modern)	Yes (historical)	Yes (historical)	Yes (historical and/or modern)
Performs population genomic analyses	Yes (genotype likelihood based)	Yes (genotype likelihood based)	Yes (genotype call based)	No	Prepares input files	No

Table S2. Comparison of PopGLen's population genomic analyses with those available in similar pipelines. GenErode and loco-pipe both perform population genomic analyses targeting similar input data as PopGLen (i.e. low coverage and/or historical samples). The below table compares the availability of different analyses in the latest versions of these pipelines with PopGLen, with the utilized software for each analysis shown in parentheses. GenErode differs from both loco-pipe and PopGLen in that it uses genotype call based analyses, whereas loco-pipe and PopGLen utilize genotype likelihoods. While PopGLen and loco-pipe both use ANGSD and related softwares to perform genotype likelihood based analyses, PopGLen currently offers some additional analyses to loco-pipe. Users can select which workflow better suits their needs, with PopGLen being especially useful to those wanting to take advantage of the integrated raw sequence processing and data filtering (i.e. repeat, mappability filtering) offered by PopGLen.

	PopGLen	***loco-pipe***	***GenErode***
Dataset filtering options	Repeats (RepeatModeler/Masker), Depth (ANGSD), Mappability (GenMap), User-provided BEDs	Depth (ANGSD)	Repeats (RepeatModeler/Masker)
LD Decay	Yes (ngsLD)	No	No
LD Pruning	Yes (ngsLD/prune_graph)	SNP thinning	Yes (PLINK)
PCA	Global only (PCAngsd)	Global (PCAngsd) and local (lostruct)	Global only (PLINK)
Admixture	Yes (NGSadmix)	Yes (Ohana)	No
Relatedness	Yes (IBSrelate, NGSrelate)	No	No
Site frequency spectrum	Yes (realSFS)	Yes (realSFS)	No
Nucleotide Diversity	Yes (realSFS)	Yes (realSFS)	No
Watterson's estimator	Yes (realSFS)	Yes (realSFS)	No
Tajima's D	Yes (realSFS)	Yes (realSFS)	No
Heterozygosity	Yes (realSFS)	Yes (realSFS)	Yes (mlRho)
F_{ST}	Yes (realSFS)	Yes (realSFS)	No
Runs of homozygosity	Yes (ngsF-HMM)	No	Yes (PLINK)
IBS matrix	Yes (ANGSD)	No	No
Allele frequencies	Yes (ANGSD)	Yes (ANGSD)	No
Analyses at subsampled seq. depth	Yes (all analyses)	No	Yes (all analyses)
Mutational load	No	No	Yes (GERP and SNPeff based)
Combined report	Yes (Snakemake)	No	Yes (Snakemake)

Supplementary References

Di Tommaso,P. *et al.* (2017) Nextflow enables reproducible computational workflows. *Nat Biotechnol*, **35**, 316–319.

Kutschera,V.E. *et al.* (2022) GenErode: a bioinformatics pipeline to investigate genome erosion in endangered and extinct species. *BMC Bioinformatics*, **23**, 1–17.

Mölder,F. *et al.* (2021) Sustainable data analysis with Snakemake [version 2; peer review: 2 approved]. *F1000Research*, **10**.

Neuenschwander,S. *et al.* (2023) Mapache: a flexible pipeline to map ancient DNA. *Bioinformatics*, **39**, btad028.

Peltzer,A. *et al.* (2016) EAGER: efficient ancient genome reconstruction. *Genome Biol*, **17**, 1–14.

Schubert,M. *et al.* (2014) Characterization of ancient and modern genomes by SNP detection and phylogenomic and metagenomic analysis using PALEOMIX. *Nat Protoc*, **9**, 1056–1082.

Zhou,Z.T. *et al.* (2024) loco-pipe: an automated pipeline for population genomics with low-coverage whole-genome sequencing. *Bioinformatics Advances*, **4**, vbae098.
